# Association between muscle strength and low back pain among middle-aged and older adults: a cross-sectional study

**DOI:** 10.1186/s12889-025-23050-2

**Published:** 2025-05-21

**Authors:** Peng Wang, Xiangjun Lu, Mohan Wen, Xu Li, Qipeng Gao, Rujie Qin

**Affiliations:** Department of Spinal Surgery, The Affiliated Lianyungang Hospital of Xuzhou Medical University/The First People’s Hospital of Lianyungang, Lianyungang, China

**Keywords:** Low back pain, Muscle strength, Older adults, CHARLS, Risk factors

## Abstract

**Background:**

There has been limited research examining the relationship between muscle strength and low back pain (LBP). Our study aims at investigating the association in middle-aged and older population in China.

**Methods:**

The dataset used in this study was derived from CHARLS Wave 3. The identification of LBP symptoms relied on self-reported data. Muscle strength was measured by grip strength (determined using a handgrip dynamometer) and chair-rising time (recorded using a stopwatch). This study employed a cross-sectional design involving 10,985 final participants. Multivariate logistic regression analysis subgroup analysis, and interaction tests were used in our study to explore the potential association.

**Results:**

A cohort of 3871 individuals (35.2%) reported a history of LBP, displaying significantly lower levels of muscle strength in comparison to the control group (grip strength, *p* < 0.001; chair-rising time, *p* < 0.001). Multivariate regression analyses revealed a significant association between decreased muscle strength and higher risk of LBP among study participants after accounting for all potential covariates (grip strength: OR, 0.974, 95% CI: 0.970, 0.979, *P* = 0.010; chair-rising time: OR, 1.056, 95% CI: 1.045, 1.067, *P* < 0.001). Restricted cubic spline models further revealed an L-shaped relationship between muscle strength and LBP, suggesting the possible potential non-linear trends in the association. The subgroup analysis indicates that this association was consistent among different groups.

**Conclusion:**

Our study revealed a significant association between weaker muscle strength and higher LBP risk, suggesting that interventions aimed at improving muscle strength may be beneficial in reducing the incidence of LBP. Further studies should address the progression of LBP and explore how muscle strength interacts with other risk factors, such as age, body mass index, and physical activity levels, to influence LBP outcomes.

## Introduction

Low back pain (LBP) is a highly prevalent and debilitating condition that affect a significant portion of the global population, particularly among older adults. It is a leading cause of disability worldwide, with an estimated 60–80% of individuals experiencing LBP at some point in their lives [1]. It encompasses a wide spectrum of pain, ranging from acute to chronic, and often overlaps with other types of musculoskeletal pain. lumbar spine is particularly vulnerable to various stressors, including mechanical overload, degeneration, and trauma, each of which can contribute to the development and persistence of LBP [2–4]. Beyond the personal suffering, LBP imposes substantial social and economic burdens, including healthcare costs, loss of productivity, and decreased functional capacity in older adults. In China, the rapid aging of the population has led to an increasing burden of LBP, exacerbating the challenges faced by the healthcare system. In addition to demographic factors, cultural attitudes toward aging and physical activity, as well as disparities in access to healthcare services, contribute to the complexity of managing LBP in China. These factors make it essential to better understand the relationship between LBP and underlying risk factors, such as muscle strength.

Muscle strength plays a crucial role in maintaining physical function and mobility. The muscles that support the lumbar spine, particularly the core muscles, are also susceptible weakness, which can lead to inadequate support for the spine, ultimately contributing to low back pain. In this study, we use grip strength and chair-rising time to assess muscle strength, both of which are widely recognized as reliable measures of muscle strength in clinical and research settings [5]. Grip strength and chair-rising time are simple yet effective indicator of overall muscle function, which has been observed as a marker of current and future health [6]. Poor handgrip strength and longer chair-rising time were strongly related to functional impairment and morbidity risk [5, 7].

Existing studies have explored the relationship between muscle strength and LBP, providing preliminary insights but suffering from several limitations. Some research indicates that lower muscle strength is associated with higher risk of LBP, suggesting that muscle weakness may contribute to the onset or exacerbation of pain. Park [8] found that low handgrip strength is closely associated with chronic low back pain in women over the age of 50 in a cross-sectional representative of the Korean general population, while Matthew [9] assessed muscle strength only in the context of physical activity, without using objective measurements in a 6,796 adults cohort. Our research aims at exploring the potential associations between muscle strength and LBP symptoms. As a condition affecting the quality of life among middle-aged and older individuals, the prevalent occurrence of LBP not only intensifies their distress but also contributes significantly to the economic and medical burdens within our society [10, 11]. Our discoveries could potentially offer insights into the creation of innovative strategies designed to mitigate the onset of LBP and improve the well-being of the older population.

## Methods

### Data resource

The dataset was sourced from CHARLS Wave 3, an ongoing cohort study conducted by the National School for Development at Peking University. The CHARLS study (https://g2aging.org/) provides longitudinal data encompassing various factors such as socio-economic and health, derived from a nationally representative sample of Chinese older individuals. The conceptual framework and measurement metrics have been aligned to be congruent with the Health and Retirement Study (HRS). It used a multistage stratified probability sampling methodology to obtain a representative sample of the population. The study encompassed 150 municipalities spanning across 28 provinces, municipal communities, and autonomous regions. Beginning with its initial data collection in 2011–2012, CHARLS has continued to gather information on population characteristics, demographics, biomedical measurements and health information every two years thereafter. The study protocol of CHARLS received ethical approval from the Ethics Committee of Peking University Health Science Center. For an in-depth exposition of the survey methodology, please refer to previous publications on the CHARLS survey design [12].

**Fig. 1:**
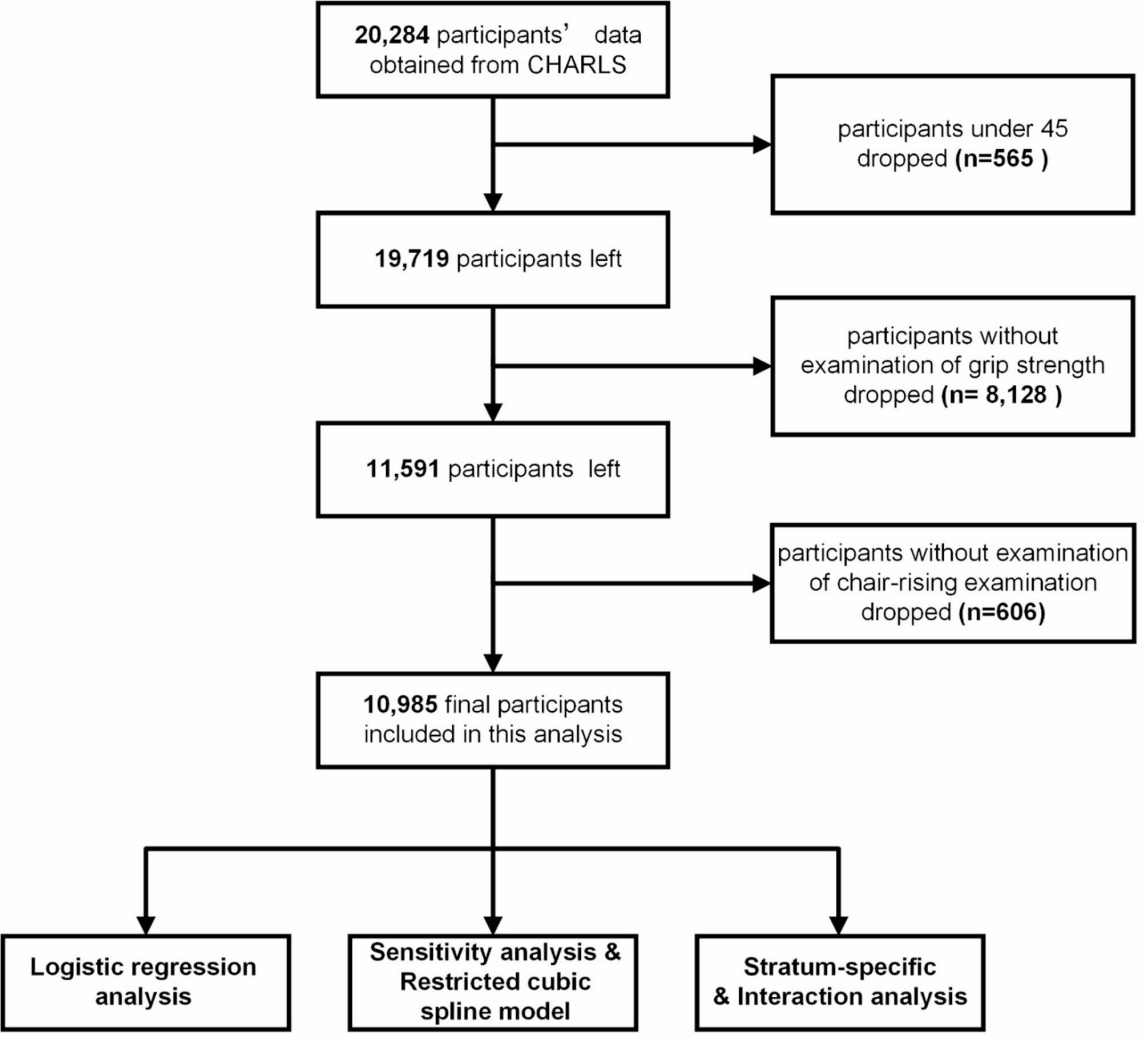
Flowchart of the participants selection

A cohort of 20,284 participants were surveyed and invited to provide consent for a venous blood sample in CHARLS Wave 3. In this cross-sectional study, participants under 45 were excluded (*n* = 565) according to the study design. Additionally, participants without grip strength (*n* = 8128) and chair-rising time examination (*n* = 606) were further excluded. Ultimately, our study enrolled a total of 10,985 individuals. Additional information regarding the criteria for participant inclusion and exclusion is detailed in Fig. 1.

### Definition of muscle strength and low back pain

At baseline, muscle strength was measured using normalized grip strength and chair-rising time. The handgrip dynamometer (YuejianTM WL-1000) was used to measure grip strength (in kilograms) twice, with the maximum value from the dominant hand recorded as the final result. Chair-rising time (seconds) was measured with a stopwatch as participants were instructed to stand up and sit down on a chair for five repetitions at their fastest pace [5].

The incidence of low back pain (LBP) was the study’s outcome. The interviewer asked the participant “On what part of your body do you feel pain? Please list all parts of body you are currently feeling pain”. Simultaneously, the interviewer identified the corresponding parts for confirmation. If the subject replied “yes” and indicated that the pain was in the lower back, the individual was classified as experiencing incident LBP [13].

### Covariates

To mitigate the influence of confounding variables, this research integrated parameters recognized for their impact on the progression of LBP. Participants were stratified into distinct groups based on various covariates: education (less than lower secondary, upper secondary & vocational training, and tertiary education); marital status (married or partnered, and separated, divorced, widowed, or never married); residential locations (rural or urban); smoking history (yes/no); drinking history(yes/no). Moreover, several disorders were identified as potential confounders based on the questionnaire” Have you been diagnosed with listed conditions by a doctor? " The study encompassed preceding medical conditions including hypertension, lung diseases, stroke, psychological disorders, arthritis, liver disease, kidney disease and gastrointestinal disorders. Multivariate imputation techniques were employed to handle missing covariate data, including variables such as age, BMI, and physical activity level using predictive mean matching (PMM) [5, 9].

### Statistical analysis

In this study, the interquartile range (IQR) and median were employed as measures of central tendency and dispersion for continuous variables demonstrating skewed distributions. Rates and percentages were utilized to describe categorical variables. The Kruskal-Wallis test was employed to assess the statistical significance for continuous variables exhibiting non-normal distributions, whereas chi-square tests were utilized to analyze associations and dependencies within categorical data [14]. Baseline data regarding the excluded population are presented in Supplementary Table 1.

Multivariate logistic regression analysis was employed to investigate the association between muscle strength and low back pain. This analysis included adjustments for covariates to minimize the potential influence of additional confounding variables on the study outcomes: Model I was adjusted solely for age. Model II was adjusted for background variables including: age groups, education, residence, marital status, smoking and drinking habits. Furthermore, Model III included additional healthy condition covariates based on Model II including hypertension, lung diseases, stroke, psychiatric conditions, arthritis, liver diseases, kidney diseases, and stomach/digestive disorders. Interaction analyses were conducted to assess the heterogeneity of the association between muscle strength and low LBP, stratified by the aforementioned covariates. Subgroup analyses were conducted employing stratified logistic regression models. The p-value for interaction was ascertained through the log-likelihood ratio test, which entailed contrasting models incorporating and omitting covariate interactions. The presence of potential non-linearity was assessed using a likelihood ratio test, comparing the linear model to a model incorporating both linear and cubic spline terms [23].

In this study, a significance threshold of 0.05 was used to assess statistical significance. All statistical analyses were conducted utilizing R version 4.4.1.

## Results

### Baseline characteristics

A total of 10,985 male participants were enrolled in the study, comprising 3871 individuals with LBP symptoms and 7,114 without. The baseline characteristics of the participants are presented in Table 1. Notably, at baseline, individuals diagnosed with LBP symptoms were observed with lower grip strength and longer chair-rising time compared to those who do not have. Concurrently, a notable discrepancy in age was observed between the LBP-afflicted cohort and the non-LBP cohort. Moreover, statistically significant disparities were also observed in gender, residency status, educational level, marital status, smoking and drinking frequence and prevalence of hypertension, diabetes, lung diseases, heart diseases, stroke, psychological diseases, arthritis, dyslipidemia, liver diseases, kidney diseases, and digestive diseases between the cohort exhibiting LBP symptoms and without.

**Table 1:** Baselines characteristics of the participants enrolled (SD: standard deviation, SD for continuous variables: P value was calculated by Kruskal Wallis rank-sum test, number (%) for categorical variables: P value was calculated by chi-square test LBP: low back pain

		Low back pain		*P*
		No	Yes	
		*n* = 7114	*n* = 3871	
Age (median [IQR])	year	60.00 [53.00, 67.00]	61.00 [55.00, 68.00]	< 0.001
Gender (%)	male	3736 (52.52)	1478 (38.18)	< 0.001
	female	3378 (47.48)	2393 (61.82)	
Grip strength (median [IQR])	(kilogram)	32.00 [25.80, 40.00]	29.00 [23.00, 36.00]	< 0.001
Chair-rising time(median [IQR])	(second)	9.37 [7.60, 11.51]	10.28 [8.35, 12.75]	< 0.001
Age class	< 50	745 (10.47)	313 (8.09)	< 0.001
	< 60	2551 (35.86)	1317 (34.02)	
	< 70	2475 (34.79)	1463 (37.79)	
	>=70	1343 (18.88)	778 (20.10)	
Education level (%)	Less than lower secondary	6182 (86.90)	3636 (93.93)	< 0.001
	upper secondary & vocational training	801 (11.26)	222 (5.73)	
	tertiary	131 (1.84)	13 (0.34)	
Marital status (%)	married or partnered	6228 (87.55)	3262 (84.27)	< 0.001
	separated divorced widowed or never married	886 (12.45)	609 (15.73)	
Residence (%)	urban	1548 (22.98)	515 (14.08)	< 0.001
	rural	5187 (77.02)	3142 (85.92)	
Smoking feq (%)	none	5036 (71.62)	2896 (75.48)	< 0.001
	<=4	187 (2.66)	138 (3.60)	
	<=10	511 (7.27)	248 (6.46)	
	> 10	1298 (18.46)	555 (14.46)	
Somking (%)	Yes	3327 (46.91)	1527 (39.48)	< 0.001
	No	3766 (53.09)	2341 (60.52)	
Drinking feq (%)	none	4508 (63.74)	2704 (70.12)	< 0.001
	less than once per day	1504 (21.27)	708 (18.36)	
	once per day	589 (8.33)	234 (6.07)	
	twice per day	336 (4.75)	150 (3.89)	
	more than twice per day	135 (1.91)	60 (1.56)	
Drinking (%)	Yes	3336 (47.13)	1645 (42.55)	< 0.001
	No	3742 (52.87)	2221 (57.45)	
Hypertension (%)	no	4639 (68.22)	2360 (63.05)	< 0.001
	yes	2161 (31.78)	1383 (36.95)	
Diabetes (%)	no	6176 (91.24)	3302 (88.60)	< 0.001
	yes	593 (8.76)	425 (11.40)	
Lung diseases (%)	no	6024 (88.16)	3028 (80.32)	< 0.001
	yes	809 (11.84)	742 (19.68)	
Heart diseases (%)	no	5842 (85.92)	2821 (75.39)	< 0.001
	yes	957 (14.08)	921 (24.61)	
Stroke (%)	no	6636 (97.03)	3605 (95.57)	< 0.001
	yes	203 (2.97)	167 (4.43)	
Psydis (%)	no	6724 (98.53)	3642 (96.68)	< 0.001
	yes	100 (1.47)	125 (3.32)	
Arthritis (%)	no	4561 (66.70)	1317 (35.11)	< 0.001
	yes	2277 (33.30)	2434 (64.89)	
Dyslipidemia (%)	no	5546 (82.99)	2871 (78.94)	< 0.001
	yes	1137 (17.01)	766 (21.06)	
Liver diseases (%)	no	6456 (94.86)	3382 (90.16)	< 0.001
	yes	350 (5.14)	369 (9.84)	
Kidney diseases (%)	no	6332 (93.04)	3128 (83.41)	< 0.001
	yes	474 (6.96)	622 (16.59)	
Digestive diseases (%)	no	5068 (74.35)	2032 (53.91)	< 0.001
	yes	1748 (25.65)	1737 (46.09)	

### Association between muscle strength and low back pain

The results of the multivariate logistic regression analyses examining the potential relationship between muscle strength and low back pain among study participants are presented in Table 2. In the unadjusted model, lower muscle strength (lower grip strength and longer chair-rising time) were significantly associated with an increased risk of low back pain (grip strength: OR, 0.970, 95% CI: 0.966, 0.974, *P* < 0.001; chair-rising time: OR, 1.066, 95% CI: 1.055, 1.077, *P* < 0.001). For Model I, the association remained significant (grip strength: OR, 0.970, 95% CI: 0.966, 0.974, *P* = 0.033; chair-rising time: OR, 1.066, 95% CI: 1.054, 1.077, *P* < 0.001). This relationship persisted in Model II (grip strength: OR, 0.974, 95% CI: 0.970, 0.979, *P* = 0.010; chair-rising time: OR, 1.056, 95% CI: 1.045, 1.067, *P* < 0.001). In the fully adjusted Model III, which incorporated all potential covariates, the association remained statistically significant (grip strength: OR, 0.974, 95% CI: 0.970, 0.979, *P* = 0.010; chair-rising time: OR, 1.056, 95% CI: 1.045, 1.067, *P* < 0.001. These findings highlight that life-related factors may affect the observed relationship between muscle strength and low back pain.

**Table 2:** Logistic regression of the relationship between grip strength/chair-rising time and risk of LBP; OR: odd ratio; CI: confidence interval; Q: quartile;

Model	grip strength		chair-rising time	
	OR (95%CI)	*P*-value	OR (95%CI)	*P*-value
Crude model	0.970 (0.966, 0.974 )	<0.001	1.066 (1.055, 1.077 )	<0.001
Model I	0.970 (0.966, 0.974 )	0.033	1.066 (1.054, 1.077 )	<0.001
Model II	0.974 (0.970, 0.979 )	0.010	1.056 (1.045, 1.067 )	<0.001
Model III	0.974 (0.970, 0.979 )	0.010	1.056 (1.045, 1.067 )	<0.001

Crude model adjust for none

Model I adjust for: age

Model II adjust for: age; gender; education level; marital status; residence; smoking frequence; drinking frequence

Model III adjust for: age; gender; education level; marital status; residence; smoking frequence; drinking frequence; hypertension; diabetes; lung diseases; heart diseases; stroke; psych problems; arthritis; dyslipidemia; kidney diseases; digest diseases; asthma

### Subgroup analysis and restricted cubic spline

**Fig. 2:**
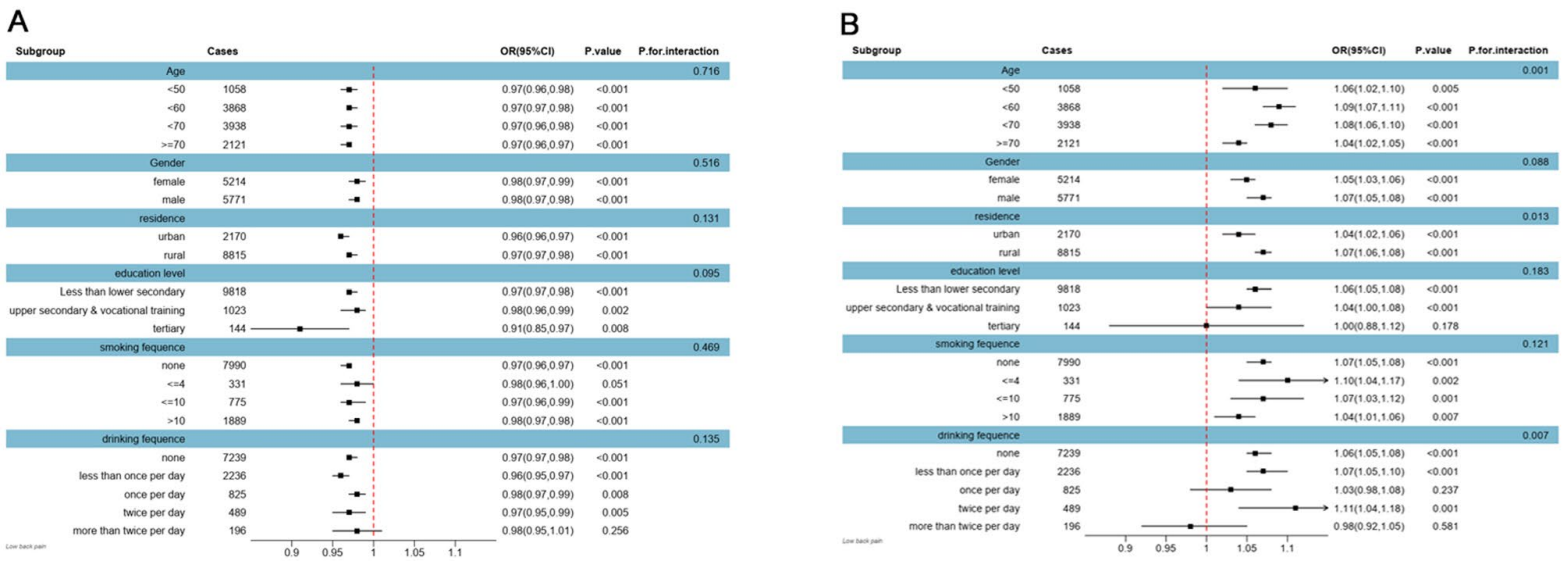
Subgroup analysis between muscle strength and low back pain; OR: odds ratio; 95% CI: 95% Confidence interval; Fig **A**: grip strength, Fig **B**: chair-rising time

**Fig. 3:**
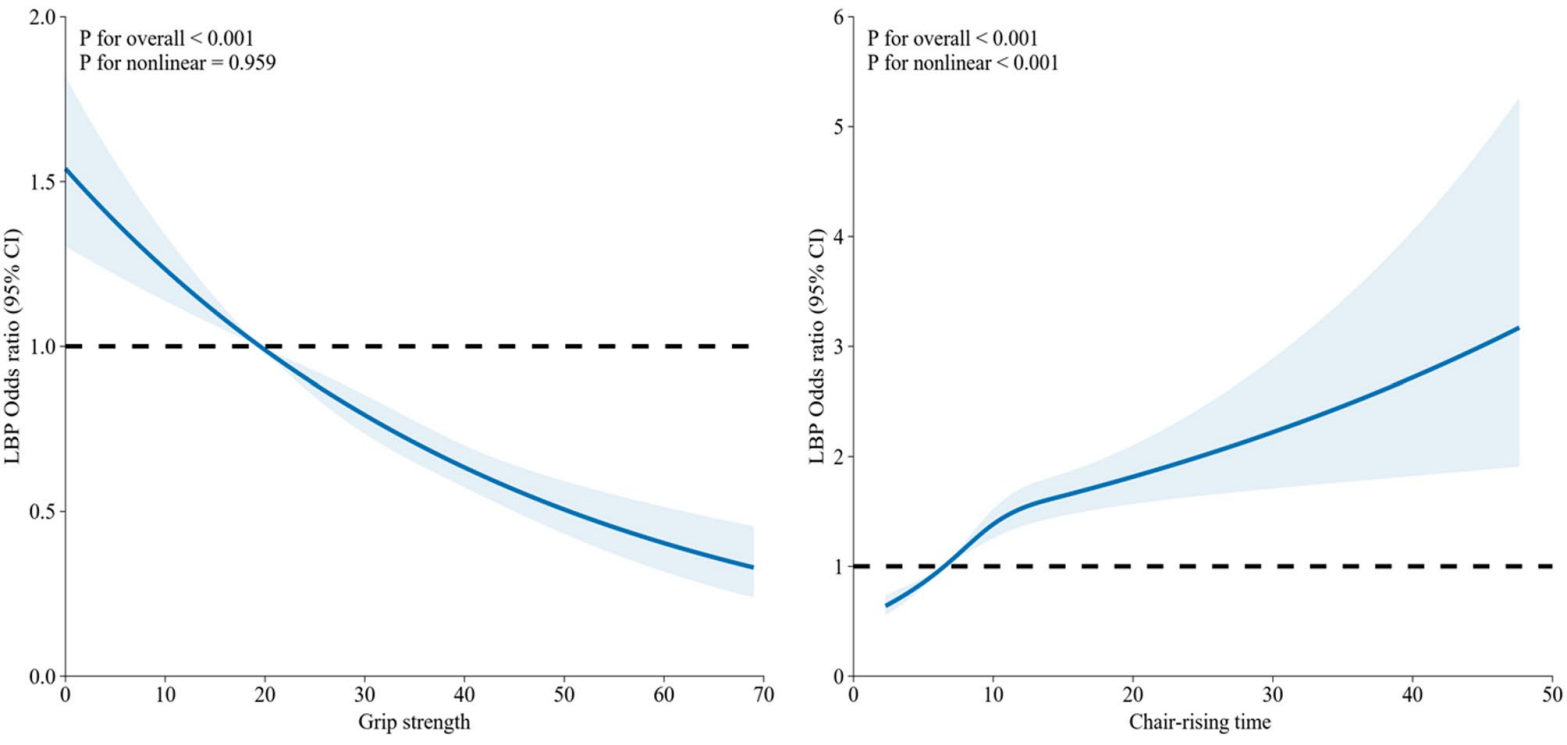
Nonlinear associations between muscle strength and low back pain in the CHARLS. Restricted cubic spline models fitted for Logistic regression models for grip strength (kilogram) and chair-rising time (second)

Based on the results of Mode III, Fig. 2 reveals the subgroup analysis of the association between muscle strength and low back pain. As shown in Fig. 2A, the correlation between grip strength and low back pain remains persist among all subgroups. As for chair-rising time, the findings from Fig. 2B indicate that this correlation of chair-rising time and low back pain remained consistent across individuals except for drinking frequence (once per day; more than twice per day). Restricted cubic spline models fitted for Logistic regression models for muscle strength (grip strength and chair-rising time) and LBP was shown in Fig. 2. L-shaped relationships were discovered between muscle strength and LBP in Fig. 3 (grip strength: p for overall < 0.001; p for nonlinear = 0.959; chair-rising time: p for overall < 0.001; p for nonlinear < 0.001). Specifically, for grip strength, the overall association was highly significant with no evidence of nonlinearity. Similarly, chair-rising time demonstrated a significant overall association and a notable nonlinear component.

## Discussion

Characterized by aging population, China is now experiencing a marked demographic expansion. Notably, there is a projected substantial rise in the proportion of individuals aged 60 and above between the years 2001 and 2031 [15]. In addition, as one of the most prevalent chronic non-communicable diseases affecting aging populations, the burden of LBP is growing worldwide. In this large-scale cross-sectional study, our study indicate that lower muscle strength is significantly associated with an increased risk of getting low back pain (LBP). This relationship was consistent across different muscle strength accessing methods, including grip strength and chair-rising time, and L-shaped relationships were also discovered.

Our results align with previous studies examining the relationship between muscle strength and LBP. However, many earlier studies were limited by small sample sizes, selection bias, which impede the generalizability of their findings [8, 16–18]. Park [8] found that low handgrip strength is closely associated with chronic low back pain, but the results was only verified in women aged over 50 years with low physical activity. Also, the study of Tanishima explored the association between sarcopenia and low back pain in a 216 local residents cohort study. The study only focused on a small population in the local community, which may introduce the selection bias. Furthermore, previous studies primarily focused on isolated muscle groups or used insufficiently comprehensive measures of muscle strength, failing to capture its full scope [17]. In contrast, our study employed a more comprehensive approach, assessing multiple muscle strength indicators. Another major limitation in past research is the failure to explore potential non-linear relationships between muscle strength and LBP. Our study contributes by considering the possibility of a L-shaped curve, where the relationship between muscle strength and LBP risk is not merely linear but may vary at different strength levels. This pattern may indicate that interventions aimed at improving muscle strength could have the most significant benefit in individuals with weaker muscle strength, where the potential for improvement is greater. These findings might be extrapolated to other populations with similar sociodemographic characteristics in other countries.

The observed association between decreased muscle strength and increased risk of getting LBP could be explained through several mechanisms. Weak muscles, particularly those supporting the lumbar spine, may lead to poor posture, reduced spinal stability, and greater mechanical strain on the lower back [10, 19]. As muscle strength declines, the ability to maintain proper alignment and absorb shock during daily activities decreases, potentially leading to higher susceptibility to pain and injury [20]. Additionally, muscle weakness may alter daily movement patterns and increase the stress on the joints and ligaments in the lower back, which contribute to the development of LBP [21, 22].

While this research was based on a nationwide investigation, it did encounter several limitations. Firstly, the study population is primarily composed of Chinese populations, which may limit the generalizability of the findings to other ethnic groups. Secondly, given the cross-sectional design of this study, establishing a precise causal relationship between muscle strength and low back pain was challenging. Moreover, we did not collect information on the progression of LBP, meaning we cannot ascertain how muscle strength might influence the long-term trajectory of LBP or its severity. However, future prospective studies and intervention-based research hold promise for providing a more comprehensive understanding of this association. Moreover, although, we adjusted for a wide range of covariates, including age, gender, BMI, and physical activity, there may still be other unmeasured factors that could influence both muscle strength and LBP risk, such as genetic factors. Also, the use of grip strength and chair-rising time may not fully capture overall muscle strength, particularly in the lower limbs and core, which are more directly relevant to low back pain. Lastly, it is important to acknowledge that certain covariates may be susceptible to recall bias.

## Conclusions

Our study revealed a significant association between weaker muscle strength and higher LBP risk, suggesting that interventions aimed at improving muscle strength, such as physical therapy and strength training, may be beneficial in reducing the incidence of LBP. Considering the rapid pace of population aging, future research should aim to explore the causal nature of this relationship through longitudinal studies and investigate the potential impact of strength training on preventing or managing LBP. Furthermore, future studies should address the progression of LBP and explore how muscle strength interacts with other risk factors, such as age, body mass index, and physical activity levels, to influence LBP outcomes.

## Data Availability

Publicly available datasets were analyzed in this study, which can be found at: https://charls.pku.edu.cn/.
